# Circulating tumour DNA analysis predicts relapse and improves risk stratification in primary refractory multiple myeloma

**DOI:** 10.1038/s41408-023-00796-9

**Published:** 2023-02-13

**Authors:** Sridurga Mithraprabhu, John Reynolds, Rose Turner, Hang Quach, Noemi Horvath, Ian Kerridge, Anna Kalff, Krystal Bergin, Jay Hocking, Flora Yuen, Tiffany Khong, Brian M. Durie, Andrew Spencer

**Affiliations:** 1 Australian Centre for Blood Diseases, Alfred Health—Monash University, Melbourne, VIC Australia; 2 Department of Malignant Haematology and Stem Cell Transplantation, Alfred Hospital, Melbourne, VIC Australia; 3 St.Vincent’s Hospital, University of Melbourne, Melbourne, VIC Australia; 4 Royal Adelaide Hospital, Adelaide, SA Australia; 5 Royal North Shore Hospital, Sydney, NSW Australia; 6 Cedars-Sinai Comprehensive Cancer Center, Los Angeles, CA USA

**Keywords:** Myeloma, Cancer genomics, Translational research

Dear Editor,

Multiple myeloma (MM), an incurable plasma cell malignancy, is the second most common form of blood cancer with a 5-year overall survival (OS) of 48.5% for newly diagnosed (ND) MM patients [1]. Prospective real-world evidence shows that 23% of NDMM patients relapse within 12 months (primary refractory) of starting bortezomib-based first-line therapy (1L) with a subsequent median OS of only 16.8 months [2–4]. Moreover, 15% of patients have a sub-optimal response (SOR) (<partial response but without progression) to 1L [3, 4]. Genomic analysis of these primary refractory patients could provide prognostic insight for the design of alternative secondary treatment approaches with 1L and/or emerging novel anti-MM drugs to improve patient outcome.

We and others have demonstrated that circulating cell-free tumour DNA (ctDNA) analysis is rapidly emerging as a robust non-invasive adjunct to bone marrow (BM) biopsy, in this spatially heterogenous disease, for comprehensive genomic analysis, therapeutic monitoring and defining the underlying biology of resistance in MM (reviewed in ref. [5]). In this study, utilising a validated and custom-designed ultradeep targeted amplicon sequencing (TAS) methodology [6], we analysed peripheral blood plasma-derived ctDNA and paired BM MM cell DNA obtained from patients enrolled in the Australasian Leukemia and Lymphoma Group (ALLG—ACTRN12615000934549) MM17 trial—a Phase II trial of response adaptive salvage treatment with carfilzomib, thalidomide and dexamethasone (KTd) for transplant-eligible NDMM patients (*n* = 50) failing bortezomib-based 1L ([7], Supplementary Methods and Supplementary Fig. 1A (SF1A)). A total of 169 samples (and paired germ-line controls from *n* = 48 patients) were subject to TAS, with specific cohorts of samples utilised for further analysis at study entry (baseline) and then sequentially to characterise and compare the dominant clones at both baseline and at relapse (Supplementary Fig. SF1A, B).

Comparison of the BM and ctDNA baseline mutational profiles indicated that 70.9% of patients had at least one shared mutation or 80.6% of patients when BM mutational data was compared with any plasma timepoint (Supplemental Data 2). *KRAS* mutations (42%; 13/31) and *ATR* mutations (29% (9/31) (Supplementary Fig. SF2A) were the most frequent in BM baseline samples while *ATR* (36.2%, 17/47) and, *FGFR3* and *ATM* mutations (27.7%, 13/47) occurred frequently in baseline ctDNA (Supplemental Data 2 and Supplementary Fig. SF2B). Chi-square test of baseline BM and ctDNA mutational profiles between patients who did (relapse; *n* = 18 ctDNA and *n* = 10 BM) or did not relapse (non-relapse; *n* = 29 ctDNA and *n* = 21 BM) on KTd identified no significant differences in the BM analysis (Supplemental Data 3), while baseline mutational ctDNA profiles revealed both *BRAF* (*P* = 0.02) and *TP53* (*P* = 0.06) mutations being more frequent in relapse patients. We next performed a mutational spectrum comparing only ctDNA variants with >1% variant allele frequency (VAF), a threshold that we have previously demonstrated to correlate with survival [6, 8] and observed an increased proportion of baseline *RAS/RAF* and *ATM/ATR/TP53* (DNA damage repair or DDR mutations) in patients who subsequently relapsed (Fig. 1A). A chi-square test for relative proportions of patients with specific ctDNA mutations (>1% VAF) also identified a statistically significant difference between relapse and non-relapse for *RAS/RAF* (22.2% vs 3.4%, *P* = 0.04) and DDR (55.5% vs 20.6%, *P* = 0.01) (Fig. 1B and Supplemental Data 3). As a result of the differences in *RAS/RAF* and DDR pathway mutations, our subsequent analyses were categorised for patients with (BM+ or ctDNA+) or without (BM– or ctDNA–) *RAS/RAF* and DDR pathway mutations.

**Fig. 1:**
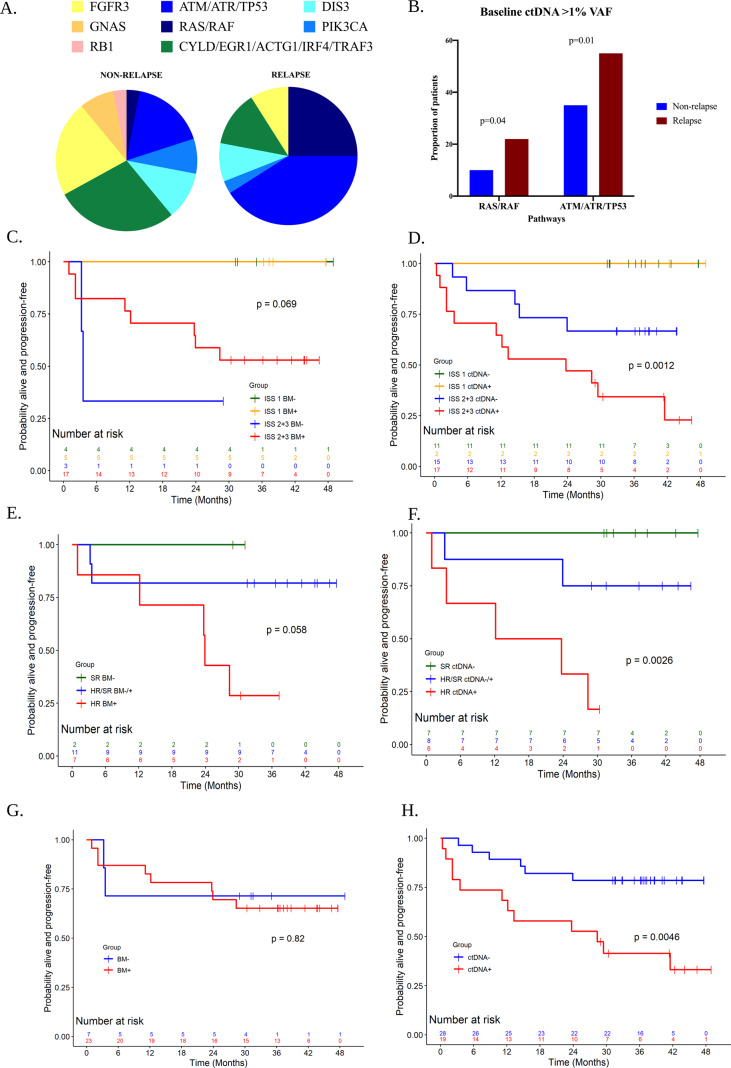
*RAS/RAF* and DDR gene mutations are associated with relapse and MM high-risk factors. **A** A representation of the ctDNA mutational spectrum (>1% VAF) in non-relapse and relapse patients indicates an increasing proportion of mutations in *RAS/RAF* (dark blue) and DDR genes (*ATM/ATR/TP53*, blue) **B** Chi-square tests proportion of *RAS/RAF* (*P* = 0.04) and DDR gene (*ATM/ATR/TP53*, *P* = 0.01) ctDNA mutations >1% VAF in patients that did (relapse, *n* = 18) or did not (non-relapse, *n* = 29) on KTd salvage therapy. **C** Kaplan–Meier survival analysis based on the presence of mutations (negative (BM–) or positive (BM+) for *ATM/ATR/TP53* or *RAS/RAF*)) and stage (ISS 1 vs ISS 2 + 3) indicated no significant differences in PFS (*P* = 0.07, Log-rank test; Median PFS in months (95% CI): NR (NA, NA) for ISS 1 and both BM- and BM+; 3.55 (3.25, NA) for ISS 2 + 3 and BM-; NR (11.14, NA) for ISS 2 + 3 and BM+). **D** Kaplan–Meier survival analysis based on the presence of mutations (negative (ctDNA-) or positive (ctDNA+) for *ATM/ATR/TP53* or *RAS/RAF*) and stage (ISS 1 vs ISS 2 + 3) indicated that advanced patients (ISS 2 + 3) with ctDNA+ had shorter PFS (*P* = 0.001, log-rank test; median PFS in months (95% CI): NR (NA, NA) for ISS 1 and both ctDNA- and ctDNA+; NR (14.49, NA) for ISS 2 + 3 and ctDNA-; 23.72 (2.17, 41.53) for ISS 2 + 3 and ctDNA+). **E** Kaplan–Meier survival analysis based on BM+ or BM- and SKY92 risk profile (HR or SR) indicated a non-significant trend towards shorter PFS (*P* = 0.06, log-rank test; median PFS in months (95% CI): NR (NA, NA) for SR and BM-; NR (3.55, NA) for HR/SR and BM–/+; 23.92 (0.99, NA) for HR and BM+). **F** Kaplan–Meier survival analysis based on ctDNA+ or ctDNA- and SKY92 risk profile (HR or SR) indicated that SKY92 HR and ctDNA positive patients had shorter PFS (*P* = 0.003, log-rank test; median PFS in months (95% CI): NR (NA, NA) for SR and ctDNA-; NR (3.25, NA) for HR/SR and ctDNA–/+; 17.94 (0.99, NA) for HR and ctDNA+). **G** Kaplan–Meier survival analysis for PFS in patients that are negative for both (BM–) versus positive for either or both DDR and *RAS/RAF* mutations in the BM on KTd therapy (BM+). No significant difference in PFS was noted (*P* = 0.822, log-rank test; median PFS in months (95% CI): NR (3.25, NA) for BM–; NR (23.92, NA) for BM+) **H** Kaplan–Meier survival analysis showed a significantly shorter PFS (*P* = 0.005, log-rank test) for patients with mutations in ctDNA+, median PFS in months (95% CI) = 28.35 (3.55, NA), compared to ctDNA-, median PFS in months (95% CI) = NR (NA, NA), on KTd). MM multiple myeloma, 1L first-line therapy, BM bone marrow, ctDNA circulating tumour DNA, ISS International Staging System, PFS progression-free survival, OS overall survival, SR SKY92 standard risk, HR SKY92 high risk, VAF variant allele frequency, KTd carfilzomib-thalidomide-dexamethasone, BM+ *RAS/RAF* and DDR-positive, BM– *RAS/RAF* and DDR negative, ctDNA+ *RAS/RAF* and DDR positive, ctDNA– *RAS/RAF* and DDR negative, CI confidence interval, NR not reached, NA not available. Figures were generated using Biorender.com.

We performed correlation of progression-free survival (PFS) and OS based on *RAS/RAF* and/or DDR ctDNA mutations when combined with recognised MM diagnostic risk factors including the International Staging System (ISS) stage (Fig. 1C, D and Supplementary Figs. SF3 and 4), SKY92 MMProfiler^TM^ risk status (Fig. 1E, F and Supplementary Fig. SF5), response to 1L therapy (Supplementary Fig. SF6), response to KTd (Fig. 1G, H), lactate dehydrogenase levels (Supplementary Fig. SF7) and cytogenetics (Supplementary Fig. SF8 and Supplementary Table ST1).

The mutational spectrum of *RAS/RAF* and DDR mutations in BM and ctDNA in MM patients categorised by ISS stage demonstrated an increase in these mutations in advanced stages (Supplementary Fig. SF3A, B, respectively). Kaplan–Meier survival analysis of groups of patients based on the presence of mutations, BM+ or BM- and stage (ISS 1 vs ISS 2 + 3) indicated that BM mutation status combined with ISS did not demonstrate any significant differences between the groups in PFS (*P* = 0.07, Fig. 1C) while ctDNA+ ISS 2 + 3 patients had significantly shorter PFS (*P* = 0.001, Fig. 1D). OS for both BM and ctDNA were not significantly different (*P* = 0.45 for BM, Supplementary Fig. SF4A or *P* = 0.12 for ctDNA, Supplementary Fig. SF4B).

We next combined ctDNA status with the SKY92 risk profile identified through BM analysis from *n* = 21 patients (Standard risk, SR = 11 and High risk, HR = 10, Supplementary Data 1). This analysis utilised equal numbers of patients for BM and ctDNA analysis. The combination of BM mutation status with SKY92 risk indicated an association with PFS (*P* = 0.06, Fig. 1E). Patients that were SKY92 HR and ctDNA+ had a significantly shorter PFS (*P* = 0.0026, Fig. 1F). Similar to ISS analysis, the OS for both BM and ctDNA did not reveal significant differences (BM OS, *P* = 0.45, Supplementary Fig. SF5A; ctDNA OS, *P* = 0.24, Supplementary Fig. SF5B). The correlation of ctDNA mutations to 1L therapy revealed an increasing proportion of *RAS/RAF* and DDR in refractory compared to sub-optimal patients (Supplementary Fig. SF6). The presence of ctDNA mutations did not correlate with LDH levels or cytogenetics (Supplementary Figs. SF7 and SF8, respectively).

We finally performed Kaplan–Meier survival comparing BM/ctDNA status of patients on KTd salvage therapy. BM analysis did not reveal any significant differences in PFS or OS (*P* = 0.82; Fig. 1G or Supplementary Fig. SF9A, respectively). However, ctDNA comparison was associated with a significantly shorter PFS (median = 28.4 months, *P* = 0.0046, log-rank test, Fig. 1H) compared to patients with no *RAS/RAF* and DDR mutations (median not reached) with a weak association for OS (*P* = 0.06, log-rank test, Supplementary Fig. SF9B).

Sequential ctDNA kinetics of relapse patients was performed to understand the biology of disease progression (Supplementary Fig. SF1B and Fig. 2). We observed that in 14/16 (87.5%) of the patients, at least one mutation at relapse/pre-relapse was already present at the start of therapy. Moreover, the mutation with the highest VAF at relapse was present at baseline in 9/16 patients (56%, patients 2, 5, 11, 13, 18, 19, 23, 40, 42; Fig. 2B, C, E, G, H, I, K, O, Q; respectively), whereas in 2 patients, the dominant mutation at relapse/pre-relapse was present at C3D1 (patients 7 and 32; Fig. 2D, M, respectively). In the remainder, a unique mutation was seen for the first time at pre-relapse, patient 12, or relapse, patients 30, 37 and 41 (Fig. 2F, L, N, P; respectively). In one patient, patient 21, no mutations were detected at relapse and only 1 mutation was present at baseline (Fig. 1J). The new mutations that emerged at relapse included *KRAS* mutations (p.G12R and p.Q61H) and mutations in *CYLD* and *GNAS* (Fig. 2).

**Fig. 2:**
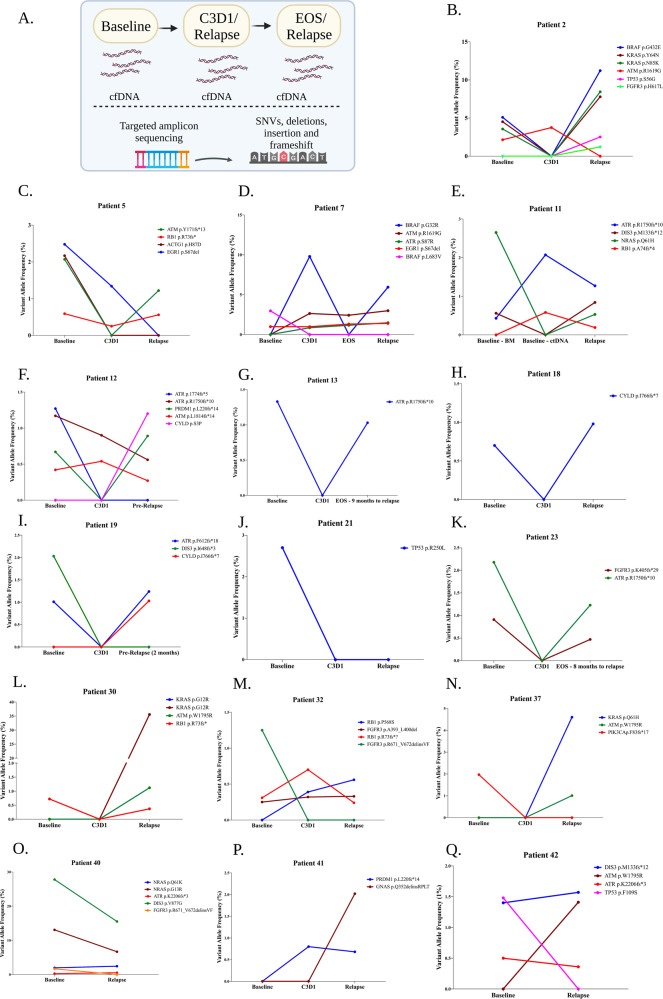
The dominant mutations at relapse are already present at the start of therapy. **A** Schematic of the analysis performed to assess the kinetics of ctDNA during treatment and subsequent relapse. **B**–**Q** Sequential ctDNA TAS analysis of 16 patients that relapsed with the VAF of the mutations present at each of the time points shown in the line graph. In 87.5% of the patients, at least one mutation at relapse/pre-relapse was already present at the start of therapy. cfDNA cell-free DNA, ctDNA circulating tumour DNA, BM bone marrow, C3D1 cycle 3 day 1, EOS end of study, VAF variant allele frequency, TAS targeted amplicon sequencing. Figures were generated using Biorender.com.

These data confirm the potential of ctDNA as a robust and risk-free methodology and support the notion that ctDNA can effectively augment BM mutational analysis for MM, particularly in the setting of large multicenter clinical trials, where ensuring the necessary quality of BM sampling is especially challenging. Our ctDNA analysis demonstrated a correlation between *RAS/RAF* and DDR pathway mutations and shortened PFS but a clear conclusion could not be made with OS due to sample size, event rate and follow-up.

The *RAS/RAF* pathway is the most frequently mutated pathway in MM [9–11] but the criteria for the selection of patients that would benefit from therapy is unclear. Our data provides a preliminary rationale for a personalised approach using *RAS/RAF* pathway inhibitors based on ctDNA mutation status. Likewise, an ineffective apoptotic response to DNA damage appeared to be a significant prognostic factor in this trial. Notably, the results from this study have reinforced previous findings, both with BM and ctDNA studies, that DDR gene mutations are markers of high risk [6, 8, 10, 12, 13], providing a context for the use of DNA-repair therapeutics in primary refractory patients. Our sequential analysis of plasma samples in patients that relapsed has provided substantial insight into the biology of disease progression in MM. The presence of high-risk secondary genetic events at relapse is known to be present at subclonal levels at diagnosis utilising BM analysis [14] and our study has provided novel evidence that this is recapitulated with ctDNA analysis. It will be interesting to ascertain if the mutations at the start of therapy in this trial were already present at the ND stage for this set of patients and could be responsible for the sub-optimal/refractory response to bortezomib-based 1L.

The limitation of our study is the modest patient cohort size, specifically in the BM cohorts. However, given that early treatment failure is evident in at least 25% of ND transplant-eligible patients, it is important to first recognise the need for comprehensive genomic analysis and this can be achieved only through incremental studies. A larger panel of MM-specific genes will also provide an improvement to the analysis cohort. This study is presented as early confirmatory data on ctDNA utility and requires validation with expanded BM and ctDNA sample cohorts from NDMM, primary refractory and eventually double refractory patients to identify the prognostic factors/biomarkers that can then be utilised for a ctDNA-based “risk” test to steer these patients to alternative therapeutic options.

## Supplementary information

Supplementary data 1

Supplemental data 2

Supplemental data 3

## Data Availability

The targeted amplicon sequencing annotated dataset utilised to perform analysis for the study are available in Supplementary Data 2 as an excel file. Statistical analysis for the Chi-Square tests is available in Supplementary Data 3 as an excel file. The raw sequencing data are part of a larger treatment-based unpublished study and are available on reasonable request from the corresponding authors.
