# Supplementary material for: Circulating tumour DNA analysis predicts relapse and improves risk stratification in primary refractory multiple myeloma
Source: Blood Cancer J. 2023 Feb 13;13(1):25. doi: 10.1038/s41408-023-00796-9 (PMC9925790; doi:10.1038/s41408-023-00796-9)
Supplement: Supplementary file 1 — Supplementary data 1 [file 41408_2023_796_MOESM1_ESM.docx]

Circulating tumour DNA analysis predicts relapse and improves risk stratification in primary refractory multiple myeloma

Sridurga Mithraprabhu*, PhD^1,2^, John Reynolds, PhD^2^, Rose Turner, MD^2^ , Hang Quach, MD^3^, Noemi Horvath, MD^4^, Ian Kerridge, MD^5^, Anna Kalff, MD^2^ , Krystal Bergin, MD^2^ , Jay Hocking MD^2,6^, Flora Yuen, MSc^2^, Tiffany Khong, PhD^1,2^, Brian M Durie, MD^6^ and Andrew Spencer*, MD^1,2^

^1^Australian Centre for Blood Diseases, Alfred Health – Monash University, Melbourne, Australia

^2^Department of Malignant Haematology and Stem Cell Transplantation, Alfred Hospital, Melbourne, Australia

^3^St.Vincent’s Hospital, University of Melbourne, Australia

^4^Royal Adelaide Hospital, Adelaide, Australia

^5^Royal North Shore Hospital, Sydney, Australia

^6^Department of Haematology, Box Hill Hospital, Melbourne, Australia

^7^Cedars-Sinai Comprehensive Cancer Center, Los Angeles, California

**Supplemental methods:**

**Study design, participants and sample collection**

Newly diagnosed multiple myeloma (NDMM) patients who were refractory, or had a sub-optimal response, based on International Myeloma Working Group criteria (IMWG), to bortezomib-based first line (1L) therapy were enrolled onto the Australasian Leukemia and Lymphoma Group (ALLG) MM17 trial (ACTRN12615000934549). The trial evaluated an intensive salvage approach utilising a combination of carfilzomib-thalidomide and dexamethasone (KTd) as re-induction (KTd x 6 cycles) and as post-autologous stem cell transplantation (ASCT) consolidation (KTd x 2 cycles followed by Td x 10 cycles). Fifty patients were enrolled onto the study and a total of 186 peripheral blood plasma and BM samples were obtained at study entry (baseline), at cycle 3 day 1 (C3D1), end of the study (EOS) and relapse (Figure 1A). IMWG uniform response criteria were used (1, 2). The study was approved by the Alfred Hospital Ethics Committee. Informed consent was obtained for all participants.

**Peripheral blood collection and processing for cell-free DNA:**

Peripheral blood plasma was collected in Streck Cell-Free BCT DNA (La Vista, NE, USA) and processed for cell-free DNA and processed. Immediately upon sample collection, the tubes were inverted to mix the blood with the preservative in the collection tube. A total of 30 millilitres (mls) of blood was collected. Plasma was separated from peripheral blood through centrifugation at 820g for 10 minutes (mins) within 24 hours of sample collection. Supernatant was collected without disturbing the cellular layer and centrifuged again at 16,000g for 10 mins to remove any residual cellular debris and stored at -80^0^ C in 1 ml aliquots for long-term storage until isolation. Frozen plasma samples were used for cell-free DNA extraction using the QIAamp circulating nucleic acid kit (Qiagen, Hilden, Germany) according to manufacturers’ instructions. Approximately, 3 mls of plasma was used for extractions. The cell-free DNA was eluted in 35 μl of buffer AVE. Subsequently, plasma cell-free DNA was quantified with a QUBIT Fluorometer 3.0 and high sensitivity DNA detection kits (Thermo Fisher Scientific, Waltham, MA, USA). The maximum input volume utilised for the QUBIT assay was 5 µl. The extracted cell-free DNA was stored at -80^0^ C until further processing.

**Peripheral blood and bone marrow collection, isolation of mononuclear and MM cells, genomic DNA extraction:**

Peripheral blood and BM aspirates were collected into 10 ml EDTA tubes and *in vitro* isolation of peripheral blood mononuclear (PBMC) and bone marrow mononuclear cells (BMMNC), respectively, was performed using SepMate^TM^ according to manufacturer’s guidelines (STEMCELL Technologies, Vancouver, British Columbia, Canada). PBMC were snap frozen as cell pellets and stored at −80 °C until further analysis. MM cell proportions in the BMMNC samples were measured with flow cytometry and MM cells were subsequently isolated using CD138+ magnetic beads(3) (Miltenyi, Bergisch Gladbach, Germany) then snap frozen and stored at −80 °C. Frozen pellets of CD138+ MM or PBMC cells were subjected to DNA extraction using a Qiagen Blood DNeasy extraction kit (Hilden, Germany), following manufacturer’s instructions. All DNA was quantified with QUBIT Fluorometer 3.0 and DNA detection kits (Thermo Scientific, Waltham, MA, USA).


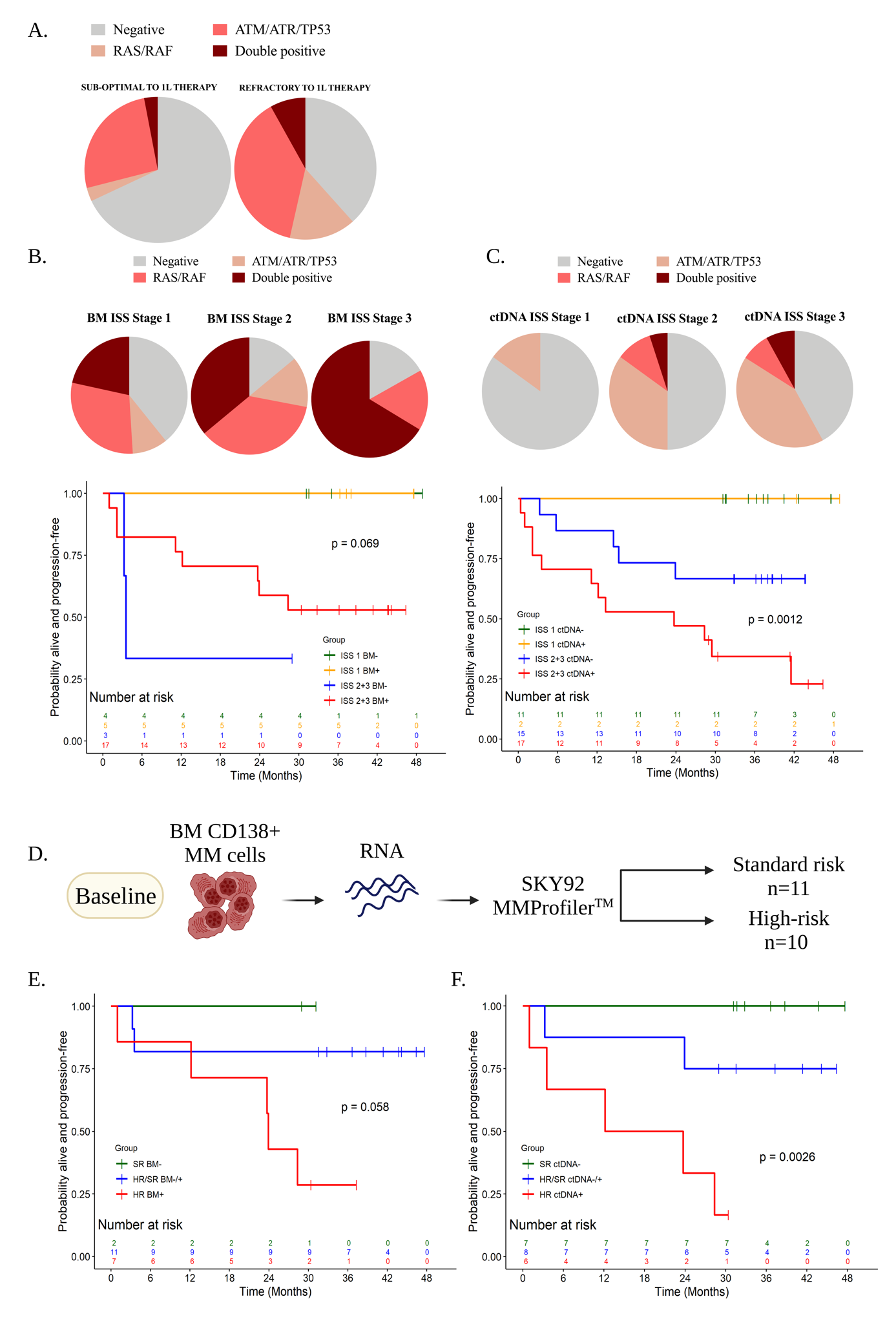
**SKY92 MMProfiler^TM^**

A total of n=21 BM samples had enough material available for assessment of SKY92 MMprofiler risk status (4). Fresh bone marrow aspirates were collected in EDTA-containing collection tubes and processed using using SepMate^TM^ according to manufacturer’s guidelines to obtain mononuclear cells followed by immunomagnetic separation of CD138-positive plasma cells using the Human CD138 microbeads (Miltenyi Biotec). RNA was extracted using the Qiagen Micro Prep kits. RNA quantity and purity were measured by Qubit RNA High Sensitivity (ThermoFisher Scientific). cDNA was prepared and biotinylated with the 3′ IVT Plus Kit and hybridization wash and stain kit (Catalog numbers 902416 and 900720, respectively; Thermo Fisher, Breda, Netherlands). Fragmented copy RNA was combined with hybridization reagents to produce hybridization cocktails hybridized to a SKY92 microarray (based on the U133 Plus 2.0 GeneChip design, ThermoFisher Scientific), and then scanned on a GCS3000Dx version 2 (Dx2 instrument; ThermoFisher Scientific). To ensure quality of the end result, quality control (QC1-QC10) acceptance criteria were evaluated.

**Current QC1 - QC10 parameters for the clinical SKY92 assay:**

|  | Item | Acceptance Criteria |
| --- | --- | --- |
| QC1 | Sample age (h) | ≤ 24 h |
| QC2 | Purity of plasma cells (after purification) | CD138 ≥ 80 % |
| QC3 | RNA yield | ≥ 100 ng |
| QC4 | Electrophoresis: rRNA 28S/18S ratio | ≥ 0.9 |
| QC5 | Electrophoresis: RNA profile | Two fragments of around 4000 nt and 2000 nt. |
| QC6 | cRNA yield (μg) | ≥ 12 μg |
| QC7 | Electrophoresis: Fragmented cRNA profile | Fragment sizes between 50 nt and 150 nt. |
| QC8 | % Present; The percentage of probe sets called Present by the Dx instrument  detection algorithm (31). | ≥ 26.6 % |
| QC9 | Scale Factor; The multiplication needed to achieve an average intensity across all  probe sets (31). | ≤ 4.0 |
| QC10 | R^2^ value for bioB, bioC, bioD and cre; The linearity of the hybridization controls (bioB, bioC, bioD and CRE) signals. | ≥ 0.545 |

If any of these 10 criteria were not met, the specimen was declared invalid and excluded from subsequent analyses (in keeping with routine SKY92 protocols). The SKY92 score was calculated as described in Kuiper et, 2012. The raw data were MAS5 normalized, log2 transformed, and mean variance normalized against a fixed reference data set of MM patient specimens. The values of 92 probe sets were multiplied by their fixed weighting factors and summed to obtain the quantitative SKY92 score, which is defined high risk when ≥0.7774, to obtain a binary outcome that is either standard risk (<0.7774) or high risk (≥0.7774).

**Targeted amplicon sequencing (TAS) and bioinformatics**

A total of 169 samples with n=48 paired germ-line controls were subject to TAS. We collected BM aspirates from 48 patients at baseline, of which only 31 were included for the TAS analysis due to poor sample quality. A total of 19 patients relapsed on the trial, of which we were able to collect baseline ctDNA and BM TAS information from n=18 and n=10 patients, respectively. For the ctDNA kinetics (Figure 2, n=16 patients) relapse ctDNA was available for n=12 patients and pre-relapse in n=2 patients. The remaining two patients had only EOS collected as the last timepoint before relapse. Plasma TAS included baseline cfDNA (n=48), C3D1 (n=44), EOS (n=32) and relapse and pre-relapse (n=12 and 2). One of the plasma samples at baseline failed sequencing and the final analysis cohort consisted of 168 samples (Figure 1B). The 22-gene custom TAS panel included *KRAS, NRAS, BRAF, ATM, ATR, TP53, CYLD, ACTG1, TRAF3, FGFR3, RB1, HIST1H1E, CCND1, MAX, PRDM1, EGR1, DIS3, PIK3CA, GNAS, IRF4, FAM46C* and *TMEM14B* using the QIAseq Targeted DNA Panel Kit for library generation and target enrichment (5). These genes were selected based on whole exome sequencing data of the most frequently mutated genes in newly diagnosed MM (6-8) and cover 95% of the genes known to be mutated in MM. Fragment size distribution and quantification of the libraries was determined with an Agilent Bioanalyzer using a DNA 7500 chip (Agilent, Santa Clara, CA, USA). Indexed sample libraries were equimolarly pooled and sequenced on an Illumina NextSeq sequencer using a NextSeq 500 High Output v2 Kit (300 cycles). The FASTQ files containing the sequencing reads were analysed using the CLC Genomics Workbench version 21 (QIAGEN). Paired end reads were trimmed and were aligned to the HG38 human genome. The read depth for each mutation identified has been provided in Supplementary data 2. Data analysis including alignment to reference genome and variant calling was carried out using QIAGEN’s CLC Genomics Workbench with the targeted amplicon sequencing workflow. Variant annotation was performed with QCI Interpret for variant calling using the default settings. Variant allele frequency (VAF), defined as the relative frequency of a mutated allele at a particular locus and expressed as a fraction or percentage of the overall allelic frequency (mutated + wild type), was derived for each sample set. Single Nucleotide Variants (SNVs) with a depth of coverage <10 in tumour or plasma samples and failed upstream filtering were excluded. The default filter settings on QCI Interpret for common genetic variants, predicted deleterious and cancer driver variants were employed. SNVs and INDELS appearing in the germ line control were excluded utilising the tumour-specific variants setting. Any variants that had <1% allele frequency were excluded for the final analysis unless the variant was present in multiple timepoints in a patient and had an allele frequency of >=0.5% in at least one of the timepoints. To validate the TAS, specific mutations were validated with droplet digital PCR on plasma samples.

**Statistical analyses:**

The trial was adequately powered for the primary efficacy endpoint (for n=50 the chance of declaring proof-of-concept for efficacy was 80% when the true ORR was 55%) but as with all clinical trials with exploratory sub-studies, the sample sizes for the sub-studies were pragmatically determined, essentially capped, by the justified sample size for the primary objective and the powers of exploratory tests for association, between non-randomizable groups and efficacy outcomes, were naturally dependent on what transpired during the trial. Statistical analyses were performed using GraphPad Prism 9 (San Diego, CA, USA) and SAS 9.4 (SAS Institute, Inc., Cary, NC, USA). Progression-free survival (PFS) was measured from the date of commencing therapy to the date of progression (IMWG uniform response criteria) or death from any cause, whichever occurred first. Overall survival (OS) was measured from the date of first commencing therapy. Survival curves, with time in units of months, were plotted to investigate the association of PFS/OS with the presence or absence of mutations and other baseline factors utilising the Kaplan-Meier method and the log-rank test for group comparisons.

**Supplementary figures:**

**Supplemental Figure 1 (SF1): Study schema and analysis cohorts**

(A) Paired BM and blood samples from patients enrolled in the ALLG MM17 trial were collected as specific time points indicated. Enriched MM (CD138+) cells were derived from the BM biopsy and cfDNA was extracted from peripheral blood plasma. Single nucleotide variants (SNVs), deletions, insertions and frameshift mutations were detected with TAS from both sources as indicated. (B) The number of samples subject to TAS for the study is presented (light yellow boxes) with information on the excluded sample numbers (blue boxes). Germline controls from PBMC from n=48 patients were included. A final analysis cohort of 168 samples (both BM and plasma) were utilised and specific analysis was performed (red boxes). The patient numbers for each of analysis is also provided. BM, bone marrow; cfDNA, cell-free DN; MM, multiple myeloma; C3D1, cycle 3 day 1 of treatment; Pre-ASCT, Pre autologous stem cell transplant; Post-ASCT, post-autologous stem cell transplant; EOS, end of study; SNVs, single nucleotide variants; TAS, targeted amplicon sequencing; SR, standard risk; HR, high risk.

**
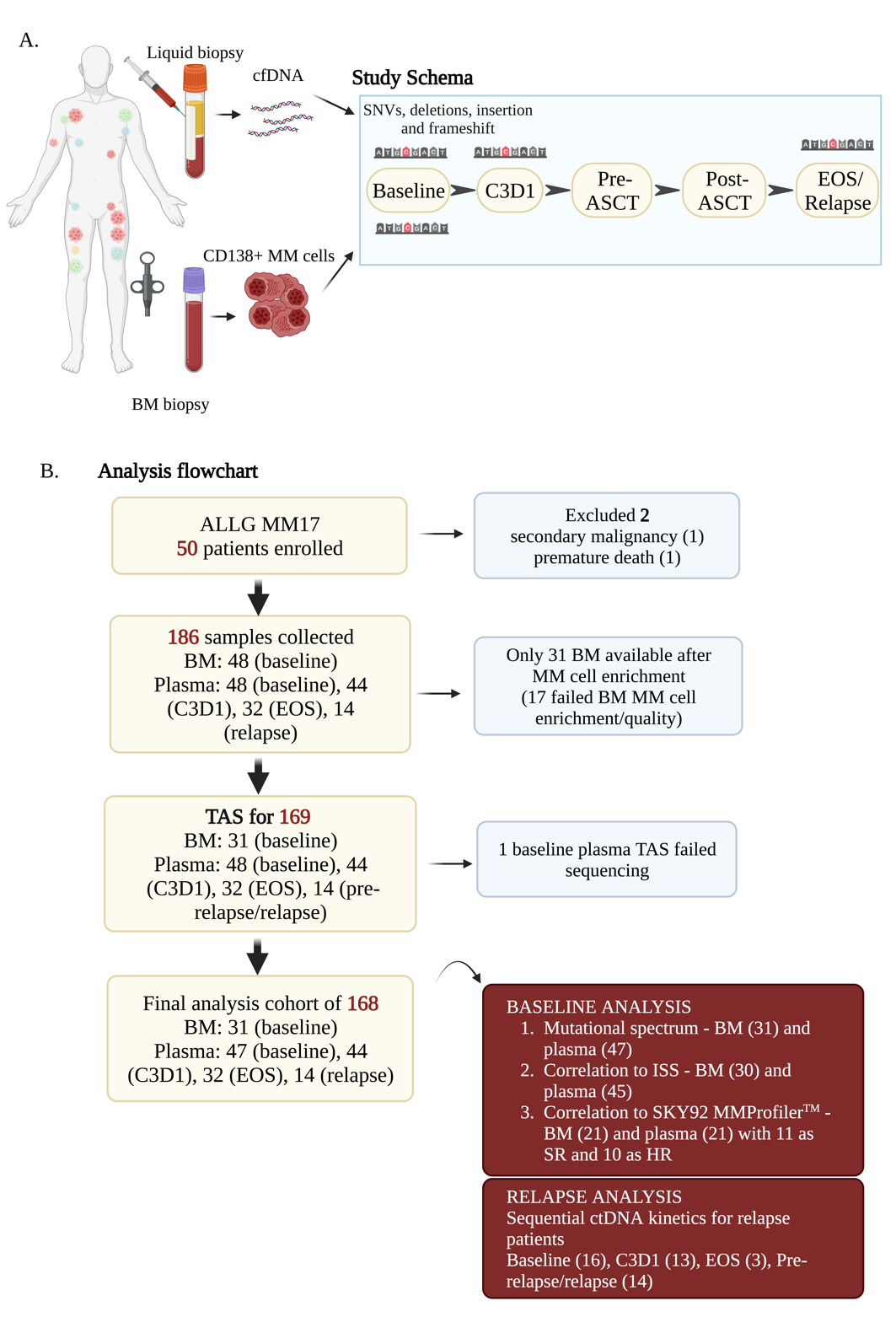
**

**SF2: Mutational spectrum MM patients at baseline reveals differences in BM and ctDNA patterns**

(A) The presence of somatic variants in specific genes identified from BM MM cell analysis (B) The presence of somatic variants in specific genes identified from ctDNA analysis. Only genes, listed as different rows, with a somatic variant detected are listed in both sub-figures. Patient numbers are indicated as columns at the top. Grey colouring indicates the absence of a somatic variant in a specific gene/patient. Blue colouring indicates the presence of a somatic variant and the number of variants is depicted with lighter shade (1 variant) or darker shade (3 variants) as indicated by the number legend on the right side of each sub-figure.

**
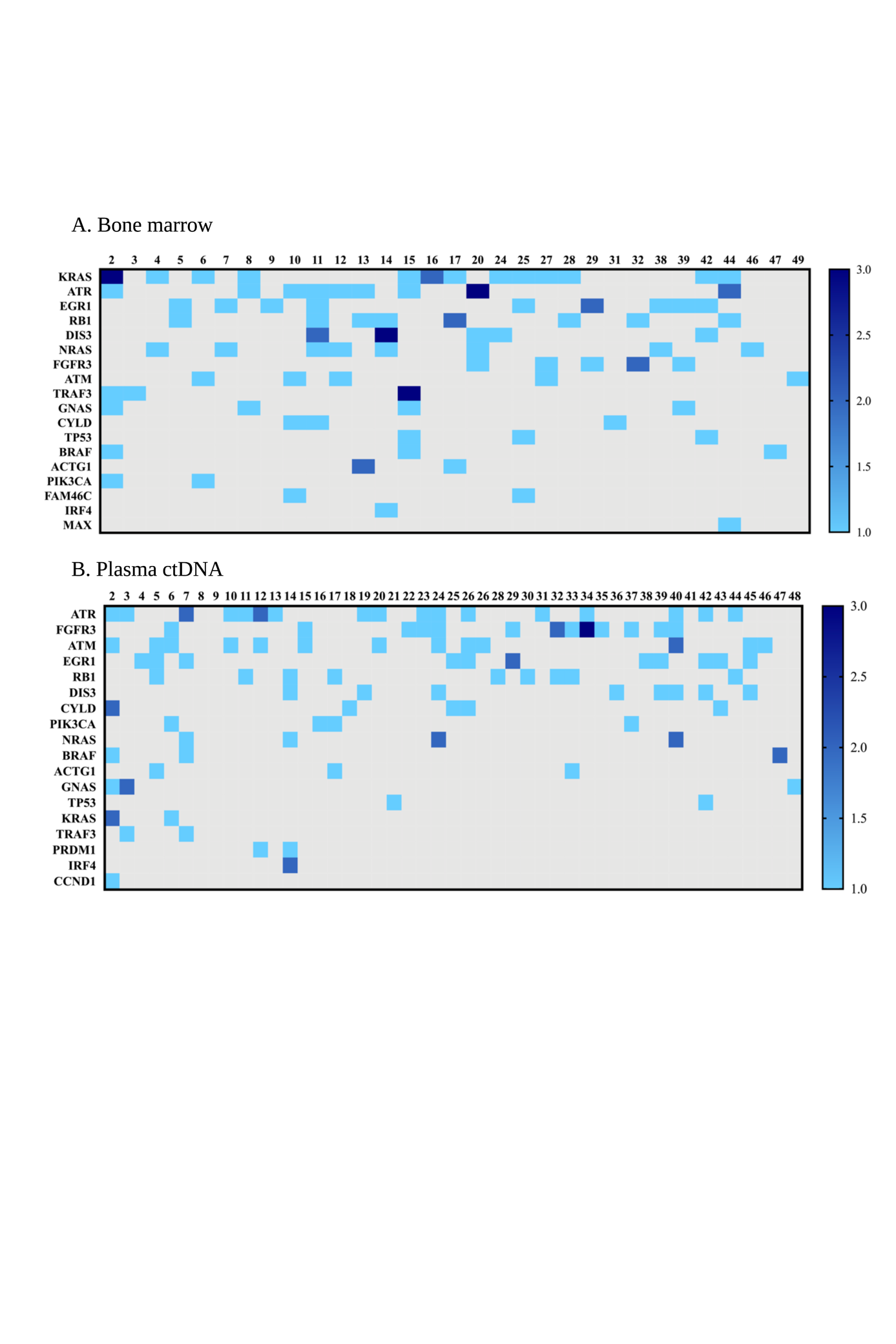
**

**SF3: Mutational spectrum of *RAS/RAF* or *ATM/ATR/TP53* in BM and ctDNA in MM patients categorised by ISS Stage**

(A) The *RAS/RAF*, *ATM/ATR/TP53* or double positive BM mutational spectrum in patients classified as ISS Stage 1, 2 or 3. An increasing proportion of patients harboured *RAS/RAF* and/or DDR mutations in the advanced stages (ISS 2 and 3)

**
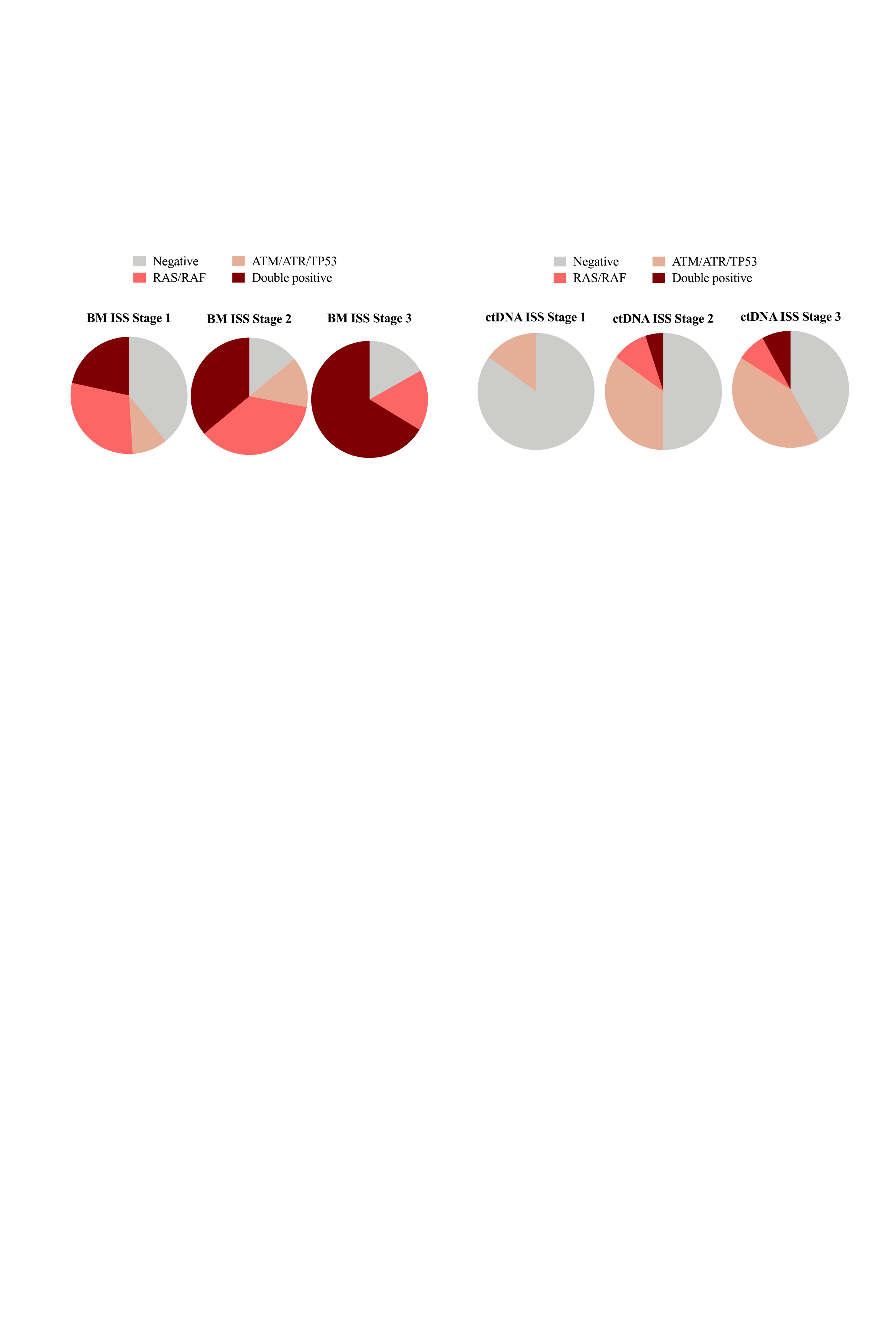
**

(B) A representation of ctDNA (>1% VAF) of *RAS/RAF* and *DDR* mutational proportions in the different ISS stages indicated an increasing proportion in ISS Stage 2 and 3 patients.

**
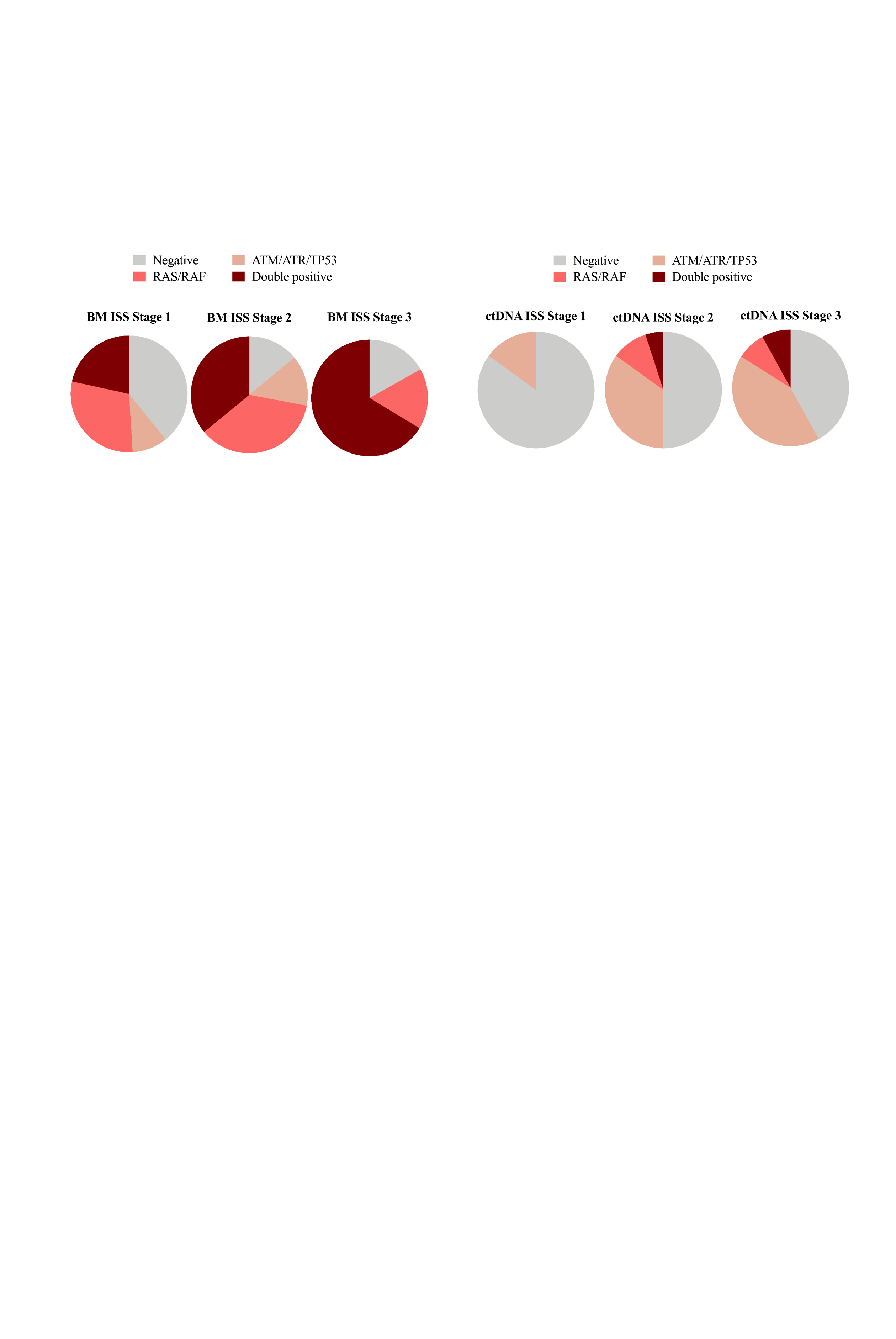
**

**SF4: Survival curves – ISS Stage and BM/ctDNA mutation status (Overall survival)**

(A) Kaplan-Meier overall survival (OS) curves comparison of International Staging System (ISS) 1, 2 or 3 with the BM mutation status (negative – neg or positive - pos for DDR and *RAS/RAF*, p=0.453).


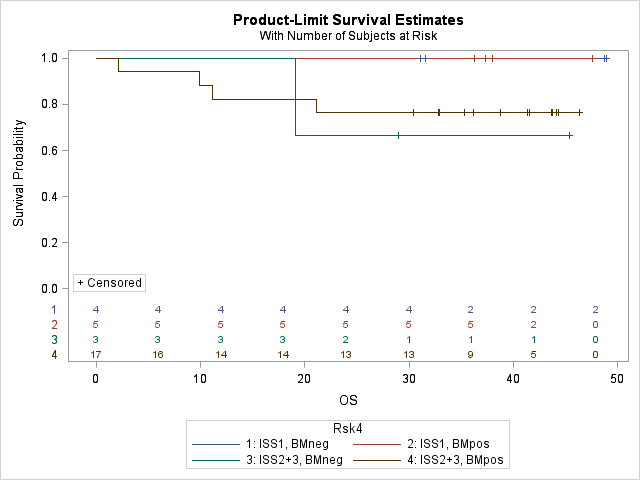


(B) Kaplan-Meier overall survival curves comparison of International Staging System (ISS) 1, 2 or 3 with the ctDNA mutation status (negative - neg or positive - pos for DDR and *RAS/RAF*, p=0.12, Log-rank test).


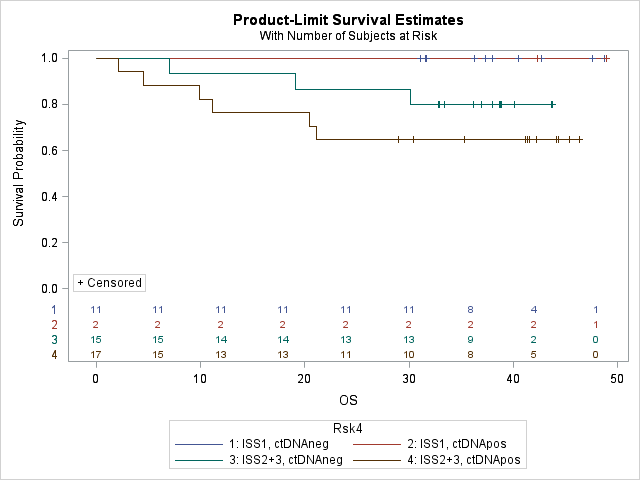


**SF5: Survival curves – SKY92 and BM/ctDNA mutation status (Overall survival)**

(A) Kaplan-Meier overall survival (OS) curves for the comparison of three groups: 1. SKY92 SR and BM-, 2. SKY92 SR BM+ or SKY92 HR BM-, and, 3. SKY92 HR BM+. Overall log-rank test p=0.450, pairwise comparison of groups 1 and 3 (p=0.221) and groups 2 and 3 (p=0.289).


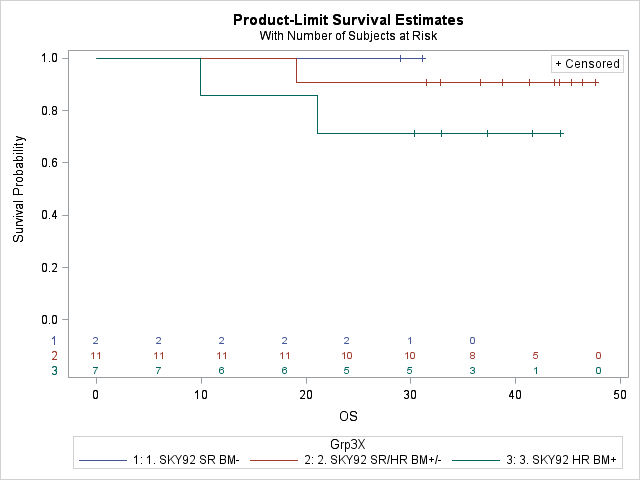


(B) Kaplan-Meier overall survival (OS) curves for the comparison of three groups: 1. SKY92 SR and ctDNA-, 2. SKY92 SR ctDNA+ or SKY92 HR ctDNA-, and, 3. SKY92 HR ctDNA+. Overall log-rank test p=0.237, pairwise comparison of groups 1 and 3 (p=0.096) and groups 2 and 3 (p=0.329).


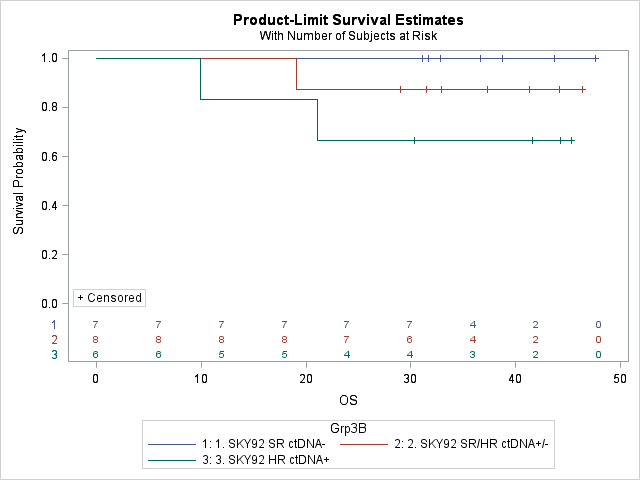


**SF6: First-line (IL) response correlation to ctDNA mutational status**

The ctDNA mutational spectrum of patients who were sub-optimal responders and patients who were refractory to 1L therapy, based on International Myeloma Working Group (IMWG) guidelines indicated an increased presence of *RAS/RAF* (3% vs 15%) and/or *ATM/ATR/TP53* (26% vs 38%) in refractory patients compared to those patients with sub-optimal response to 1L therapy.

**
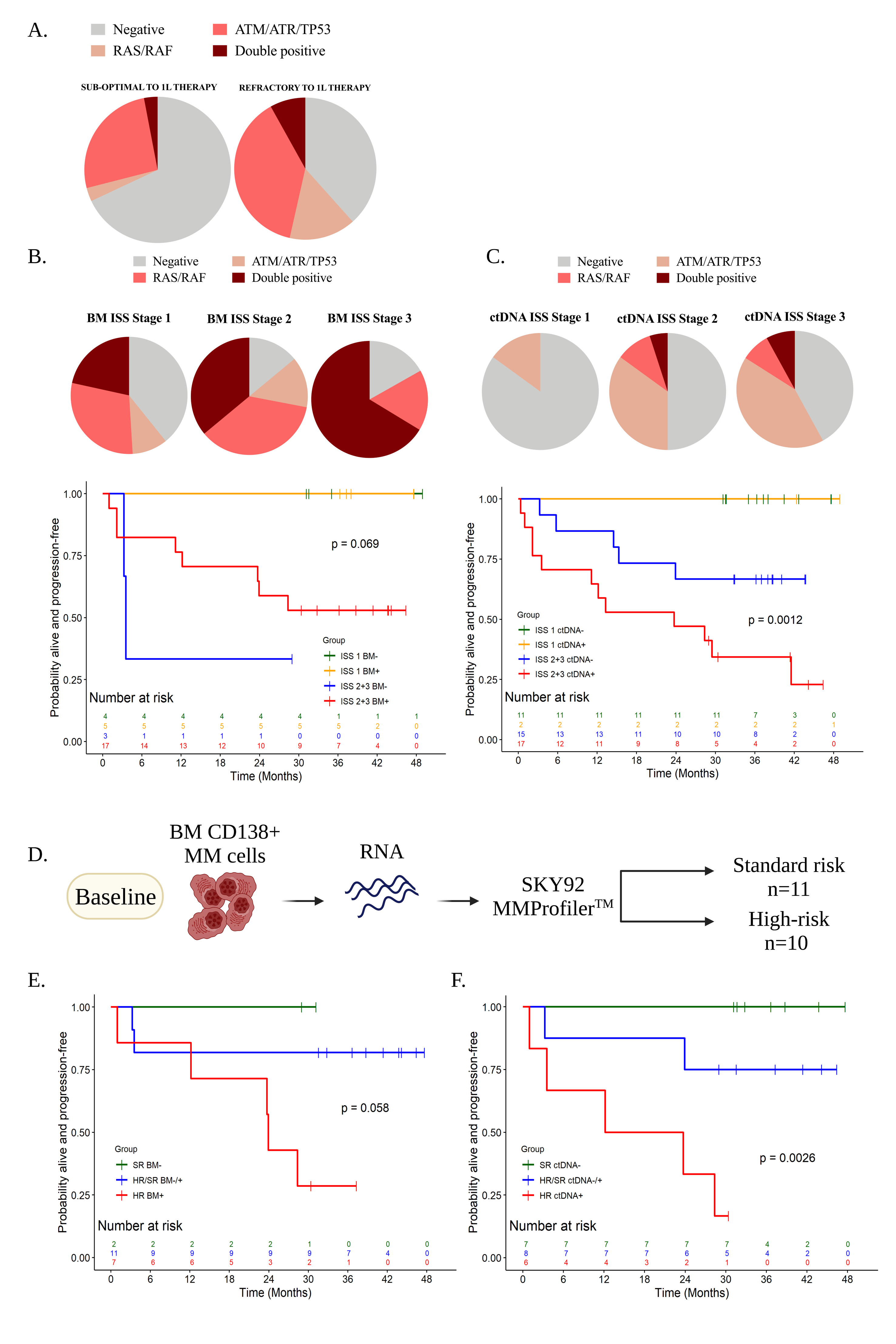
**

**SF7: LDH levels and ctDNA**

Comparison of lactate dehydrogenase levels (U/L) between ctDNA positive (*RAS/RAF* and/or DDR) and negative patients. No significant differences were noted.


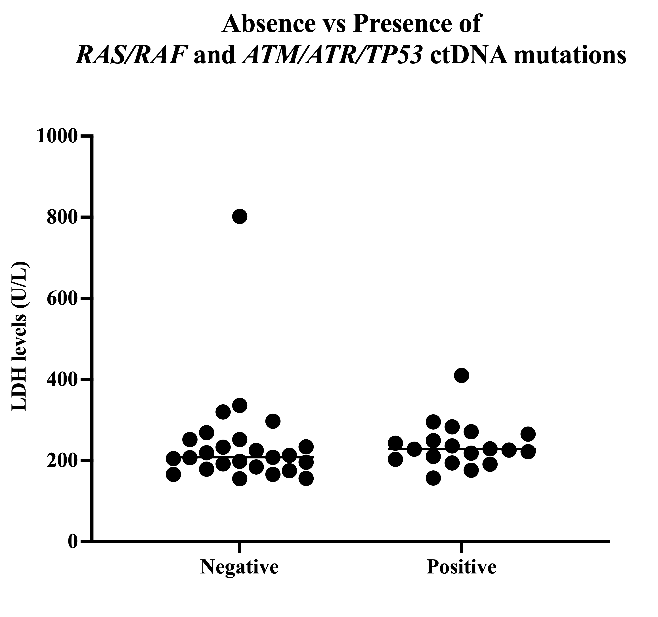


**SF8: Correlation with karyotype/FISH results**

Proportions of patients based on the availability of cytogenetics (FISH/Karyotype) and the absence or presence of ctDNA mutations. No marked differences were noted.

**SF9:** **Survival curves – Response to KTd and BM/ctDNA mutation status (Overall survival)**

(A) Kaplan-Meier survival analysis for OS in patients that are negative for both versus positive for either or both DDR and *RAS/RAF* mutations in the bone marrow. No significant difference in OS (p=0.823)


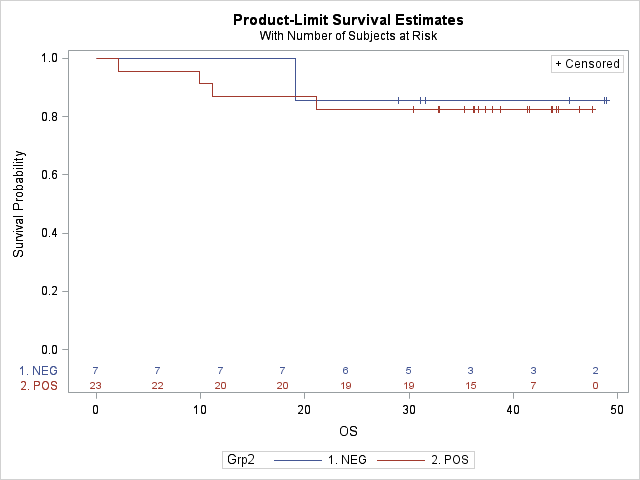


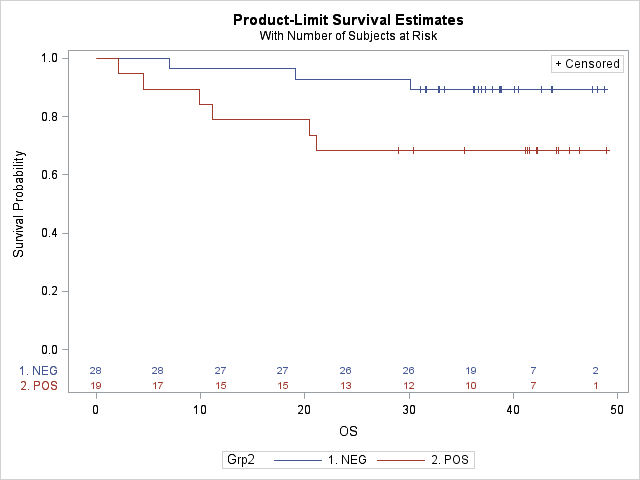
(B) Kaplan-Meier survival plots indicates a shorter association in OS (p=0.06, Log-rank test) for patients with ctDNA mutations in either pathway or both pathways (*RAS/RAF* and/or DDR).

**Supplementary table 1 (ST1): Cytogenetics/FISH/Karyotype information and BM/ctDNA availability**

| Patient | Cyto/FISH | BM | ctDNA |
| --- | --- | --- | --- |
| 1 | Hyperdiploidy | Excluded | Excluded |
| 2 | Hypodiploidy, monomy 13 and gain of 1q | Available | Available |
| 3 | Normal | Available | Available |
| 4 | Trisomy 17 | Available | Available |
| 5 | Complex Karotype. Deletion ch 13 | Available | Available |
| 6 | Indicating the presence of either trisomy of 14q32 or of IGH translocation other than the t(4;14) | Available | Available |
| 7 | Normal | Available | Available |
| 8 | Trisomy of 14p32 of the presence of an IGH translocation other than the t(4;14) | Available | Available |
| 9 | Indicates the presence of trisomy of 4p16 or of an FGFR3 translocation other than the t(4;14) | Available | Available |
| 10 | Normal | Available | Available |
| 11 | Complex Hypodiploid Karotype. 1p-, 1q+, MYC Translocation | Available | Available |
| 12 | Normal | Available | Available |
| 13 | Normal | Available | Available |
| 14 | 17p-. IGH Translocation | Available | Available |
| 15 | 1q+ | Available | Available |
| 16 | Hypodipliod. Complex Karotype. Del 13 | Available | Available |
| 17 | Normal | Available | Available |
| 18 | No Report | Not available | Available |
| 19 | Not Evaluable | Not available | Available |
| 20 | IGH translocation other than the t(4;14) and/or gain of 14q32 | Available | Available |
| 21 | Trisomy of 4p16 | Not available | Available |
| 22 | Normal | Not available | Available |
| 23 | Complex Karotype. 17p- | Not available | Available |
| 24 | Normal | Available | Available |
| 25 | Hyperdiploid. 17p-. IGH Translocation | Available | Available |
| 26 | Not Evaluable | Not available | Available |
| 27 | Normal | Available | Available |
| 28 | Normal | Available | Available |
| 29 | Normal | Available | Available |
| 30 | Tetrasomy of 1q | Not available | Available |
| 31 | Normal | Available | Available |
| 32 | Hypodiploid. 1p-, 1q+, del13 t(4;14) | Available | Available |
| 33 | No Report | Not available | Available |
| 34 | Either trimosy of 14q32 or of IGH translocation other the t(4;14) | Not available | Available |
| 35 | Normal | Not available | Available |
| 36 | Trisomy of 4p16 | Not available | Available |
| 37 | Hypodiploid. t(11;14) | Not available | Available |
| 38 | No Report | Available | Available |
| 39 | Trisomy for 4p16 or of a translocation involving 4p16 | Available | Available |
| 40 | No Report | Not available | Available |
| 41 | Hypodipliod. Complex Karotype. | Not available | Available |
| 42 | Hypodiploid. Del 13, 17p- | Available | Available |
| 43 | No Report | Not available | Available |
| 44 | Normal | Available | Available |
| 45 | Complex Karotype. 1q+, del13, t(14;16) | Not available | Available |
| 46 | Hypodipliod. Complex Karotype. | Available | Available |
| 47 | 1p-, 1q+, t(4;14) | Available | Available |
| 48 | No Report | Not available | Available |
| 49 | t(11;14) | Available | Not available |
|  | No Report | Excluded | Excluded |

**References**

1. Durie BG, Harousseau JL, Miguel JS, Blade J, Barlogie B, Anderson K, et al. International uniform response criteria for multiple myeloma. Leukemia. 2006;20(9):1467-73.

2. Rajkumar SV, Harousseau JL, Durie B, Anderson KC, Dimopoulos M, Kyle R, et al. Consensus recommendations for the uniform reporting of clinical trials: report of the International Myeloma Workshop Consensus Panel 1. Blood. 2011;117(18):4691-5.

3. Mithraprabhu S, Kalff A, Chow A, Khong T, Spencer A. Dysregulated Class I histone deacetylases are indicators of poor prognosis in multiple myeloma. Epigenetics. 2014;9(11):1511-20.

4. Kuiper R, Broyl A, de Knegt Y, van Vliet MH, van Beers EH, van der Holt B, et al. A gene expression signature for high-risk multiple myeloma. Leukemia. 2012.

5. Mithraprabhu S, Hocking J, Ramachandran M, Choi K, Klarica D, Khong T, et al. DNA-Repair Gene Mutations Are Highly Prevalent in Circulating Tumour DNA from Multiple Myeloma Patients. Cancers (Basel). 2019;11(7).

6. Morgan GJ, Walker BA, Davies FE. The genetic architecture of multiple myeloma. Nat Rev Cancer. 2012;12(5):335-48.

7. Walker BA, Boyle EM, Wardell CP, Murison A, Begum DB, Dahir NM, et al. Mutational Spectrum, Copy Number Changes, and Outcome: Results of a Sequencing Study of Patients With Newly Diagnosed Myeloma. J Clin Oncol. 2015;33(33):3911-20.

8. Walker BA, Morgan GJ. The genomic features associated with high-risk multiple myeloma. Oncotarget. 2018;9(84):35478-9.
